# Kyasanur Forest Disease: A Comprehensive Review

**DOI:** 10.7759/cureus.65228

**Published:** 2024-07-23

**Authors:** Srilekha N, Venkataramana Kandi, Sri Ram G, Jayashankar CA, Harshitha A, Akshay AS, Challa Kapil, Pratyusha S Palacholla

**Affiliations:** 1 Internal Medicine, Vydehi Institute of Medical Sciences and Research Centre, Bangalore, IND; 2 Clinical Microbiology, Prathima Institute of Medical Sciences, Karimnagar, IND; 3 General Medicine, Vydehi Institute of Medical Sciences and Research Centre, Bangalore, IND

**Keywords:** kyasanur forest disease virus (kfdv), epidemiology, ecology, ticks, vector-borne infections, pathogenicity, zoonotic, india, kyasanur forest disease

## Abstract

Vector-borne microbial diseases are ubiquitous, and their management remains elusive. Such diseases with zoonotic potential result in public health challenges requiring additional control and preventive measures. Despite their cosmopolitan presence, vector-borne infections are neglected due to their endemicity in specified geographical regions. The Kyasanur forest disease (KFD) caused by the Kyasanur forest disease virus (KFDV) is among such diseases transmitted through ticks and localized to India. Despite its prevalence, high transmissibility, and potential to cause fatalities, KFDV has not been given the deserved attention by the governments. Further, KFDV circulates in the rural and wild geographical areas threatening infections to people living in these areas with limited access to medical and healthcare. Therefore, physicians, healthcare workers, and the general population need to understand the KFDV and its ecology, epidemiology, transmission, pathogenesis, laboratory diagnosis, and control and prevention as described comprehensively in this review.

## Introduction and background

Kyasanur forest disease (KFD) is a zoonotic viral tick-borne illness caused by the Kyasanur forest disease virus (KFDV). KFDV emerged first in 1957 from the Kyasanur forest in Karnataka, India [1]. Monkeys are primarily affected by KFDV. However, other mammalian species including humans are infected by the KFDV. The hard ticks of the genus *Haemaphysalis *transmit the KFDV. Ectoparasitic ticks carry various pathogenic organisms, transmitting bacterial, viral, and rickettsial diseases. Tick distribution is influenced by climate and environmental factors worldwide. Ticks are expanding their range, and their bites can go unnoticed due to painlessness, emphasizing the need for tick bite prevention [2]. 

KFD has two clinical phases including the primary phase wherein patients suffer from hematological abnormalities like leukopenia, thrombocytopenia, and elevated liver enzymes. In the secondary phase, the patients develop neurological complications [3]. Multiple human infections are documented annually in South India, with a fatality rate of 2-10% [4]. The presence and spread of ticks are influenced by various factors, including biotic and abiotic conditions, climate, and interactions with disease-causing pathogens [5]. KFD manifests within 2-8 days of infection wherein the patients present with symptoms like high fever, headache, fatigue, and bleeding from the mouth, nose, and stomach. Red and itchy eyes may be seen, while other symptoms remain elusive [6]. Despite sharing pathogenesis with other flaviviruses, a comprehensive understanding of KFD's pathogenic mechanism and host response is limited [7,8].

Efforts to discover an effective remedy for KFD have been unsuccessful, and the existing vaccine's efficacy is limited [9]. Mitigating the impact of KFD involves prevention, early detection, and supportive care. Deforestation, urbanization, and changing climate patterns contribute to the emergence of KFDV [10]. The KFD outbreak's complexity involves the transmission from monkeys to humans and the continued maintenance of the virus [11]. Since its discovery, KFD cases have been regularly detected each year resulting in considerable morbidity and mortality. KFDV was found to involve new geographical areas outside the endemic region from where it was first discovered suggesting the potential for its spread. This is evident by an increase in the number of infections since 2011. Considering its zoonotic capabilities and the occurrence of the simultaneous sylvatic (wild) cycle, one health approach was suggested as a potential strategy to control and prevent the spread of KFDV [12].

This review explores the evolution and biology of KFDV, including the epidemiology, impact of environment, clinical course, pathophysiology, laboratory diagnosis, treatment, and prevention and control of KFD.

## Review

Virus

**Figure 1:**
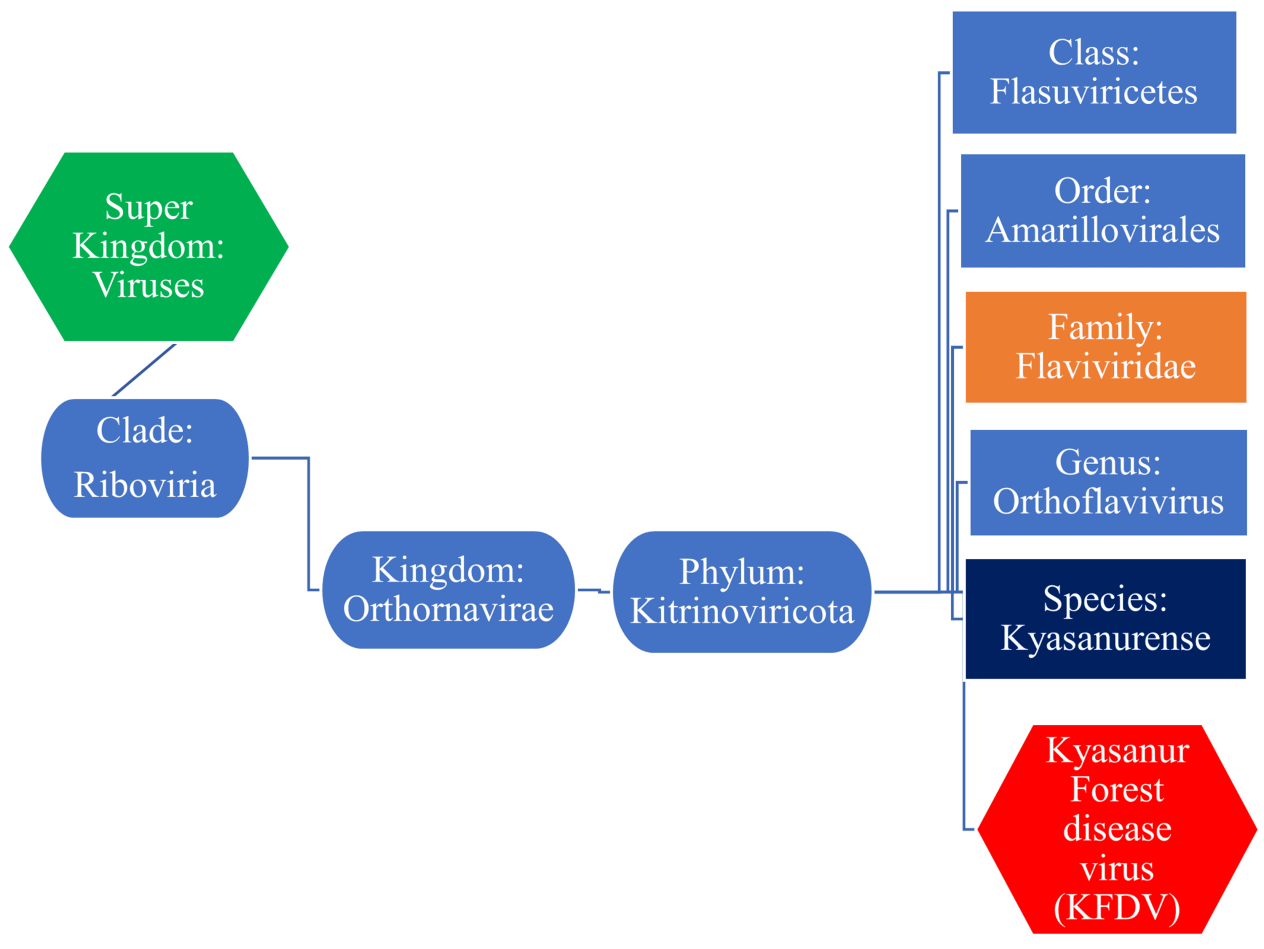
Taxonomical position of the KFDV KFDV: Kyasanur forest disease virus Image Credit: Venkataramana Kandi

The KFDV is a ribonucleic acid (RNA) virus consisting of about 11 kilobytes (kb), single-stranded (ss), positive-sense (+) RNA genome. The KFDV belongs to the family of *Flaviviridae *and the genus *Orthoflavivirus* (Figure 1).

The viral genome encodes a polyprotein consisting of three structural proteins (capsid-C, transmembrane-M, and envelope-E) and seven non-structural (NS) proteins (NS1, NS2a, NS2b, NS3, NS4a, NS4b, NS5) [13]. Despite being discovered more than 70 years ago, KFDV and the disease are not sufficiently understood, and the pathogenesis of KFD is not thoroughly described. This is evident by the unavailability of specific treatments to prevent and manage the disease [11,14]. Whole genome analysis revealed the genetic characteristics of the KFDV, a virus affecting humans and monkeys. A subgroup of KFDV strains emerged in 1980 and spread to non-endemic states including Goa and Maharashtra. A genetic site, 123 A/T, near the envelope protein-dimer interface, shows evidence of adaptive evolution and potential functional implications in the viral multiplication in the host cells. These findings emphasize the need for surveillance and improved vaccination strategies to control the spread of KFDV [13].

Host

KFD frequently causes infections that result in fatalities among two South Indian monkey species including *Macaca radiata* (red-faced bonnet macaque) and langurs (gray langur) belonging to the genus *Semnopithecus *[3]. The KFDV also amplifies in other primates like black-faced hanuman langur (*Presbytis entellus*). Monkeys have been identified as important reservoir hosts for KFDV. However, the KFDV-infected monkeys often succumb shortly after infection unable to withstand the onslaught of the virus. High death rates were reported in monkeys infected with KFDV, especially in the endemic regions of India [8]. Domestic animals like cattle do not effectively amplify the KFDV but can serve as reservoirs for the primary vectors. The role played by cattle in the transmission of KFD is unclear. Recent studies have identified various small forest mammals capable of maintaining and transmitting KFDV to ticks, emphasizing the significance of co-feeding among ticks on a single host for efficient transmission [15].

The vector

The primary vector for KFDV transmission is the hard-bodied tick *Haemaphysalis *(*H*.) *spinigera*. Other tick species that could transmit the KFDV include *H*.* tortures*, *H*. *turturis*, and *H*. *kinneari*. Soft ticks of the *Ornithodoros *genus also can transmit the virus. These ticks have been found in 11 states and seven union territories of India. *H*. *spinigera *was identified as the most predominant tick with the highest transmissibility of KFDV [16]. KFDV RNA was surveyed in the ticks involved in the disease in Kerala, India. This study found viral RNA in 5.35% of the ticks. *Haemaphysalis *(92.72%) was the predominant vector species followed by *Amblyomma *species [17]. In a global geological survey, different species of ticks were reported to harbor and transmit KFDV along with other viruses like Bourbon virus, Dhori virus, Powassan virus, Omsk hemorrhagic fever virus, Colorado tick fever virus, Crimean-Congo hemorrhagic fever (CCHF) virus, and Heartland virus [18].

Transmission cycle

**Figure 2:**
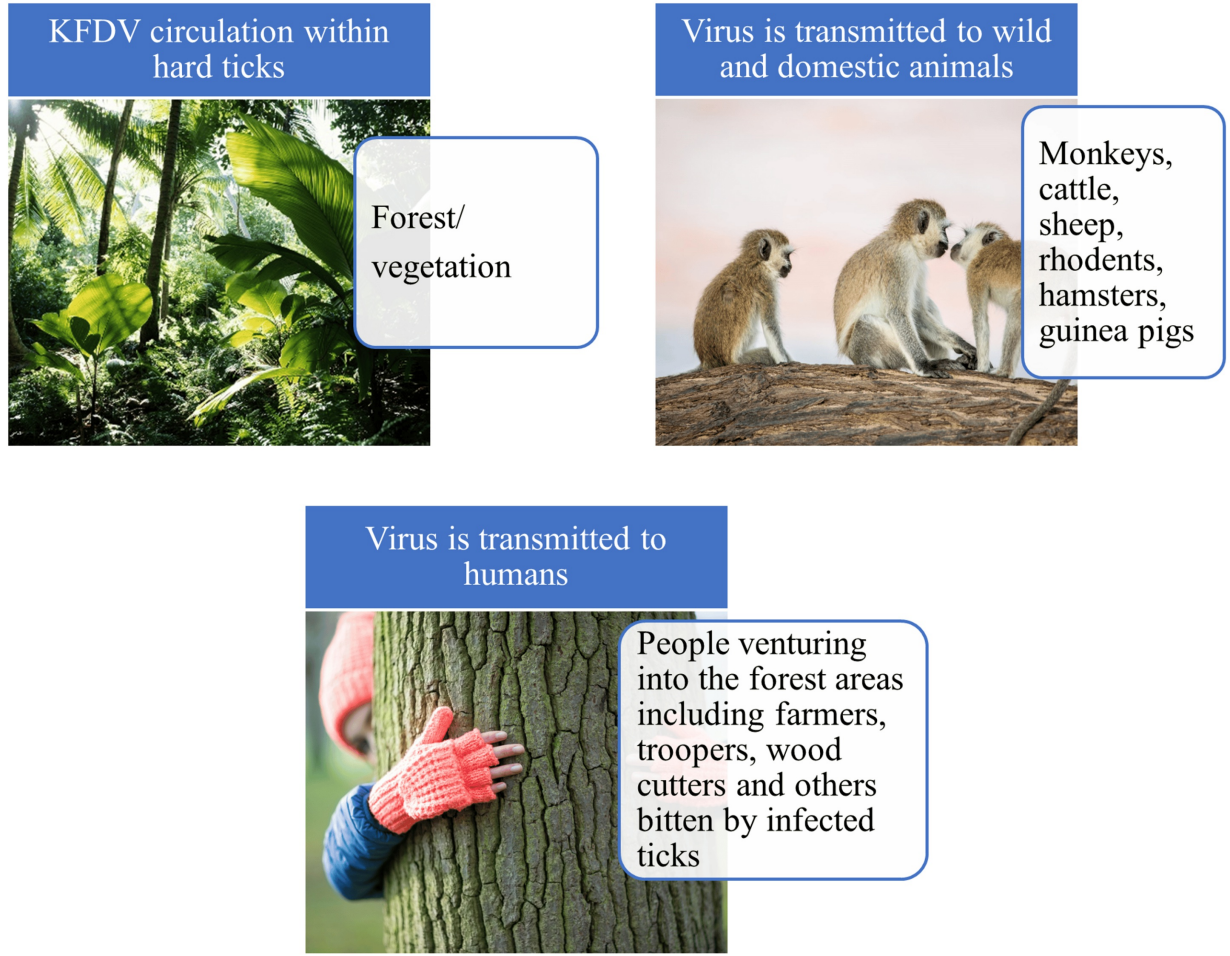
The ecological features of the KFDV KFDV: Kyasanur forest disease virus Image Credit: Venkataramana Kandi

The transmission cycle of KFDV involves a complex interaction between the virus, tick vectors, and mammalian hosts as shown in Figure 2.

Although the primary vector is *H*. *spinigera*, other tick species like *Rhipicephalus *were also identified as potential vectors. The virus is transmitted from infected animals to ticks during their blood meal wherein the virus replicates in the gut and other organs. The virus is subsequently transmitted to susceptible hosts during the next blood meal of the infected tick. Small mammals including rodents, hamsters, and guinea pigs could serve as amplifying hosts, as evidenced by the high viral loads that potentially infect ticks. Larger mammals act as maintenance/reservoir hosts and typically harbor lower virus loads in their blood, limiting transmission to ticks. Human infection occurs through tick bites, primarily in tick-infested forest areas. Direct human-to-human transmission has not been reported [15].

Role of the environment

Deforestation in the Western Ghats of India has resulted in the loss of forest cover, creating new zones for the emergence of KFDV [18]. The destruction of habitats also has led to an increase in rodent populations which act as hosts for the ticks that transmit KFDV [19]. The dense vegetation in the forests provides a favorable environment for the tick vectors [20]. Deforestation and agricultural expansion have escalated human-wildlife interaction and the risk of KFDV transmission [21]. Monkeys and rodents have been identified as key elements in introducing and spreading KFDV to humans [22]. Climate change has a transient role in KFD epidemics, affecting the expansion of hosts, reservoirs, and vectors [23]. Changes in temperature and rainfall patterns influence the abundance of KFDV inhabiting hosts and vectors [24]. Global warming is expected to increase the prevalence of vector-borne diseases like KFD [25]. Continuing investigations are required to understand the potential spread of KFDV to new regions and address possible future health crises [24]. According to a recent study from India that assessed the role of temperature and rainfall in the development and spread of ticks and its relationship with the infection rates, the tick population showed slow multiplication when the temperatures exceeded the favorable range of 20-31°C. However, the tick populations flourish following precipitation/monsoon season enabling their spread [26]. Although KFD was first discovered in Shimoga, Karnataka, India, and was initially recognized as monkey fever, the virus has been spreading across other states bordering Karnataka like Kerala, Goa, Maharashtra, and Tamil Nadu. A bio-climatic model called Maximum Entropy (MaxEnt) was used to assess the role of environmental temperature in the spread of KFD. This study identified that ticks favored a temperature between 25.4°C and 30°C [27].

Epidemiology

**Figure 3:**
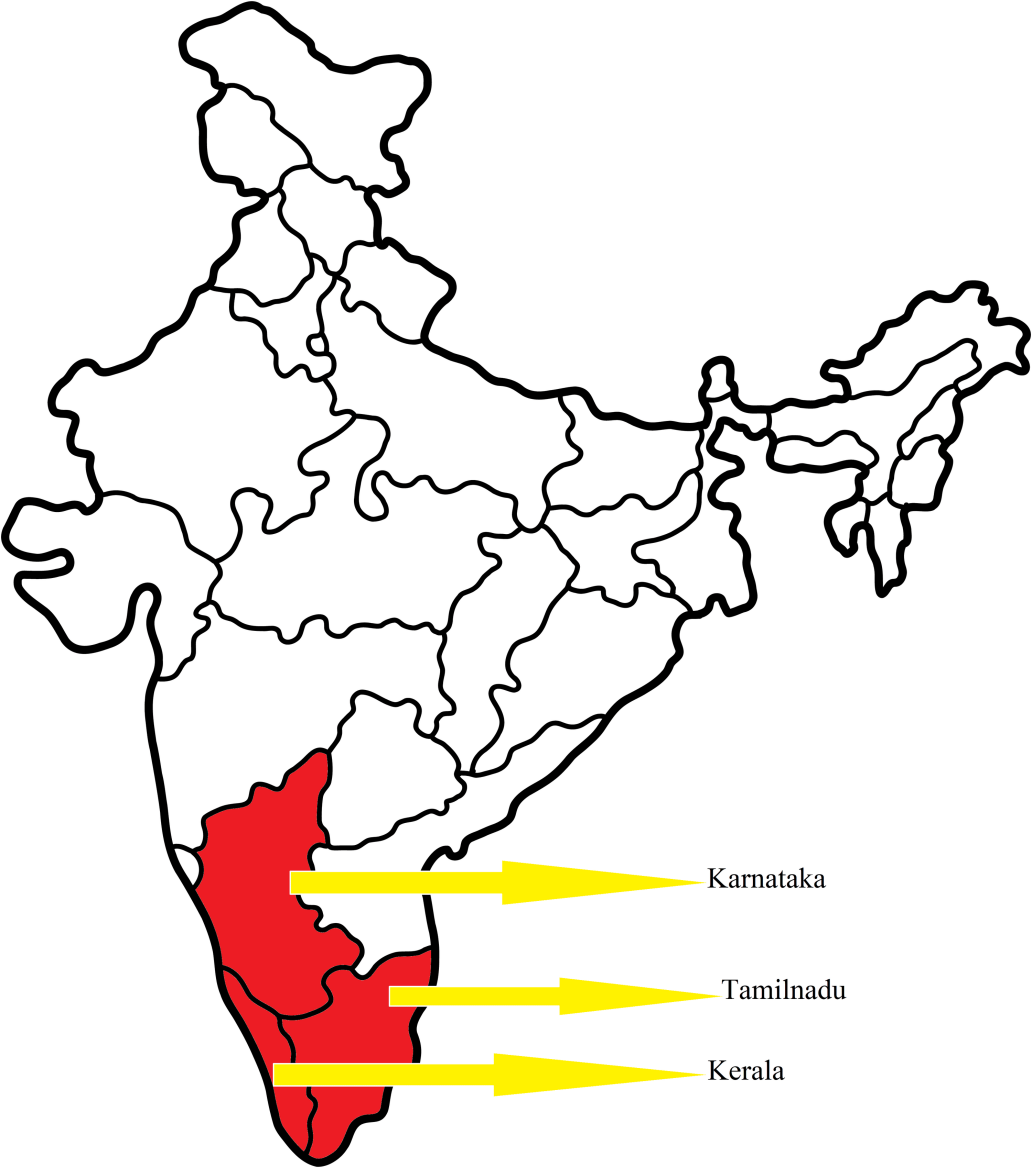
Epidemiology of the KFD KFD: Kyasanur forest disease Image Credit: Venkataramana Kandi

According to the Centers for Disease Control and Prevention (CDC), Atlanta, United States, KFD is endemic to Karnataka, Kerala, and Tamil Nadu states of India [28] (Figure 3).

KFD cases have also been reported from other non-endemic regions like some parts of Goa, Maharashtra, and Western Ghats. Additionally, serological evidence demonstrating the presence of antibodies against KFDV was observed in people living in the states of West Bengal, Gujarat, and Andaman and Nicobar Islands of India [29]. These reports signify the potential of the virus to spread and involve larger geographical regions. Despite the availability of few studies, the exact numbers of KFD cases in India have not been available. Previous reports indicated approximately 10000 KFD cases in India, with nearly 400-500 cases yearly between 1957 and 2017 [8]. No particular sex predisposition was noticed with 52% of males and 48% of females being infected. There was no significant difference in the mortalities among males (54%) and females (46%), and most infections occurred in the age group of 15-64 years (78%) as per the data provided by the Program for Monitoring Medical Emerging Diseases. However, higher death rates among men (70%) than in women (30%) were seen in other studies [30]. These reports highlight the urgent need for more proactive surveillance mechanisms.

Clinical course

Early accounts of KFD primarily focused on its hemorrhagic symptoms and overlooked notable indicators of neurological illness [31]. Initially, KFD was misidentified as a viral hemorrhagic fever based on the observations among KFDV-infected patients who revealed hemorrhagic lesions, lung consolidation, and gastrointestinal bleeding, especially in fatal cases [32]. However, further investigations revealed similarities between KFD and other acute febrile illnesses caused by tick-borne encephalitis (TBE) viruses, distinguishing them from typical meningoencephalitis-causing viruses [33]. Clinical manifestations of KFD varied from a general febrile illness to a fatal hemorrhagic disease exacerbated by dehydration [31]. Subsequent studies identified neurological symptoms such as confusion, disorientation, nuchal rigidity, and tremors in KFD patients [34].

Analysis of cerebrospinal fluid and postmortem examinations did not provide clear indications of meningitis or encephalitis [35]. Research on KFDV infection suggested a possible link with the Louping ill virus, which affects sheep but can also potentially affect humans [6]. Comprehensive reports published after the first KFD outbreak described the disease's clinical features, including a two-phase illness. In the first phase, after an incubation period of 3-8 days, patients develop symptoms like fever, chills, headache, and myalgia [29]. In the second phase, the disease resembles TBE but with additional hemorrhagic manifestations. The initial phase demonstrated a sudden-onset fever, chills, headache, myalgia, lymphadenopathy, conjunctival suffusion, and petechial hemorrhage. Some patients experienced a period of remission before entering the second phase which is characterized by neurological symptoms such as severe headache, tremors, rigidity, photophobia, mental confusion, and impaired vision. Generalized convulsions were associated with poor outcomes in some patients [8,29].

The clinical course of KFD appears to be complex and augments further substantiating examinations. Animal experiments in guinea pigs, ferrets, and hamsters demonstrated no severe viral infection. Nevertheless, tissue examination of hamsters for KFDV RNA revealed the presence of viral RNA in the lungs, liver, kidney, and spleen [36]. Radiological evaluation of patients suffering from KFD showed evidence of neurological involvement in the first phase (11.5%) and the second phase (36.4%) of the infection, with the latter showing additional brain involvement and complications. Evidence of focal infarcts was common in the first phase, and involvement of cerebellar and leptomeningeal regions was noticed during the second phase of infection [37]. Neurological involvement in the first two weeks of infection correlated with the worst clinical outcomes. Low erythrocyte sedimentation rates, urinary sediments (66%), and albuminuria (60%) were some of the key laboratory findings among KFD patients along with marked leukopenia, moderate thrombocytopenia, and elevated liver enzymes [36].

KFD pathogenesis

To increase the understanding of KFD pathogenesis, alternative animal models were being explored that could depict the disease pattern among humans. Laboratory experiments in pigtailed macaques (*Macaca nemestrina*) showed that post-infection, the animals developed lymphopenia, thrombocytopenia, and elevated liver enzymes, a consequence that was noticed among humans during natural infection. Additionally, the virus was noted in the gastrointestinal system of the pigtailed macaques, a feature commonly seen among infected humans [38].

Predisposing factors for infection with the KFDV include people living in low socioeconomic conditions, people with poor access to land, people living in poverty, religio-cultural presumptions, and lack of information transfer about the disease by the ancestors [28,39,40].

**Figure 4:**
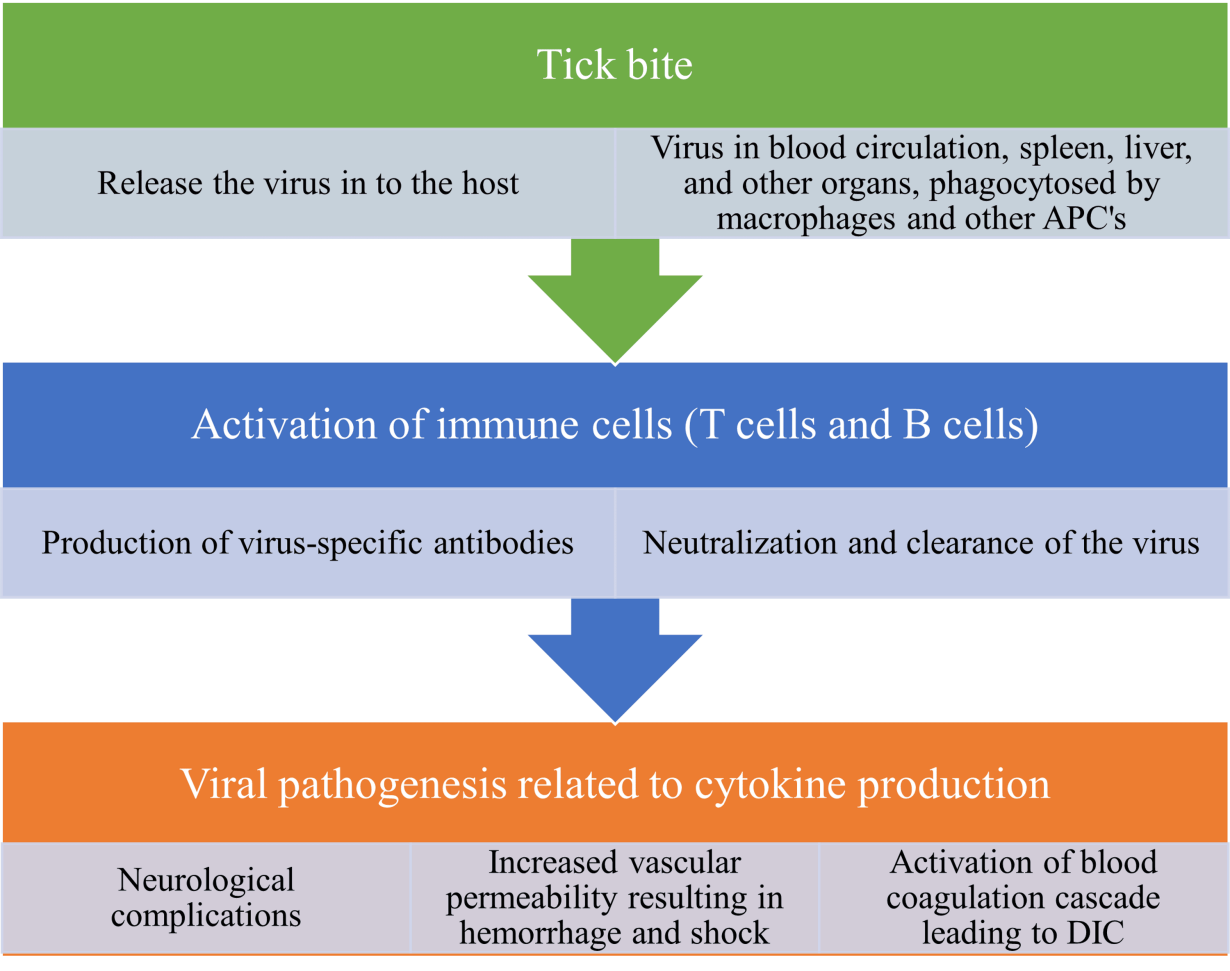
Pathophysiology and complications of the KFD KFD: Kyasanur forest disease; APCs: antigen-presenting cells; T cell: T lymphocyte; B cell: B lymphocyte; DIC: disseminated intravascular coagulation Image Credit: Venkataramana Kandi

The virus deposited into the human skin through tick bites is initially engulfed by the macrophages and other antigen-presenting cells (APCs). The APCs transport the virus to different organs of the body. Additionally, the APCs stimulate the T (thymus) cells and B (bone marrow) cells which then initiate the production of T-lymphocyte subsets like CD4+T lymphocytes and antibodies, respectively. The antibodies react with the viral antigens and result in the neutralization and clearance of the virus. The viral entry into the host also stimulates the production of cytokines which could potentially result in complications like disseminated intravascular coagulation (DIC), hemorrhagic manifestations, and neurological complications. A schematic representation of the pathophysiology of KFD is presented in Figure 4.

Laboratory diagnosis of KFD

Diagnosis of KFD is challenging due to its similarity to other endemic illnesses, such as dengue fever and malaria. Laboratory testing is crucial for confirmation, but this can be difficult in remote areas lacking appropriate laboratory facilities. The Indian Council of Medical Research (ICMR) and the National Institute of Virology (NIV), Pune, India, have developed a point-of-care (POC) testing kit to diagnose KFD. It utilizes a battery-powered polymerase chain reaction (PCR) analyzer. The Truenat KFD test revealed high sensitivity and specificity, with a notable limit of detection (LoD) of the virus in clinical specimens. Besides, nested reverse transcription (RT)-PCR and TaqMan-based real-time RT-PCR targeting specific viral gene sequences for the early diagnosis of KFD are available. Also, immunoglobulin (Ig) M antibody capture (MAC) enzyme-linked immunosorbent assay (ELISA) aids in detecting recent/acute infections. These tests enhance the diagnostic capabilities against hemorrhagic viruses in India and assist in implementing appropriate control measures and managing KFD patients [41,42]. The handling of patient samples and performing laboratory tests like RT-PCR, real-time RT-PCR, and IgM ELISA is recommended in a biosafety level 2 (BSL-2) laboratory, while virus isolation necessitates a BSL-4 laboratory [43].

A study of the KFD outbreak in the Sindhudurg district of Maharashtra, India, showed that patients were positive for viral RNA approximately 21 days after infection. Additionally, this study found evidence of anti-IgM antibodies among infected patients that were detectable for approximately four months, and anti-IgG antibodies were detectable even a year after the initial symptoms [44].

KFD patients were enrolled and followed up from the day of onset of illness. Specimens including blood, throat swabs, feces, and urine samples were collected from the patients. The results of this study revealed that the viral RNA was evident in 100% of blood samples during the first four days. However, the positivity rates were zero at the end of the first week. A small percentage (14%) of patients showed viremia for up to 15 days after the onset of symptoms/illness. The study also found evidence of viral RNA in feces and urine specimens during the early stages of infection [45]. Considering the significance of timely diagnosis in managing KFD patients, a recent study evaluated the efficacy of a POC diagnostic assay. This study positively assessed and compared the performance of rapid PCR-based assay with the conventional real-time RT-PCR approach [43].

Envelope domain III (ED III) antigen-based indirect human IgM ELISA was useful in diagnosing acute cases of KFD [46]. A one-step reverse transcription loop-mediated isothermal amplification (RT-LAMP) assay was suggested as a cheap alternative method with similar sensitivity and specificity as conventional PCR in diagnosing KFD [47]. Viral metagenomics wherein different viral etiologies could be de-identified from the single sample collected from suspected patients was suggested to identify the infection among symptomatic patients [48].

Risk assessment

High-risk groups and factors for acquiring KFD include monkeys and humans residing in forested endemic areas, areas with increased plantations, dense moist evergreen woodlands, and high cattle density. Occupational exposure among forest workers, plantation workers, and farmers who can come in contact with ticks is considered a high-risk population. Several challenges could potentially interfere with the effective management and control of KFD in India. Some problems identified include improper surveillance methods, lack of diagnostic tools, delay in diagnosis, limited vaccine availability, and inadequate personnel training [49].

Newer technological advancements including machine learning applications like event-based surveillance (EBS) and transfer learning (TL) techniques along with other models including Extreme Gradient Boosting (XGB) and long-/short-term memory models were being explored to predict human KFD outbreaks/infections, especially in the resource-limited settings [50].

Tick control and management

Treating the forest floor with gamma-hexachlorocyclohexane and using tick repellents like N,N-diethyl-meta-toluamide (DEET), picaridin, oils like oil of lemon eucalyptus (OLE), para-menthane-diol (PMD), citronella, soybean, peppermint, and other essential oils are effective methods for tick control [51]. Other tick management strategies include personal protection, landscape modifications, host management, and area application of acaricides. Integrated actions include mowing grass, removing leaf litter and brush, discouraging rodents, relocating firewood and bird feeders, injecting ivermectin into cattle, managing pets, trimming vegetation, utilizing hardscape/xeriscape, creating borders, and widening trails [52].

Treatment and vaccination

KFD does not have a specific antiviral drug therapy. Supportive management involves proper hydration and circulation/hemodynamic maintenance through intravenous fluids, colloids, or blood products. However, research has shown that sofosbuvir and its active metabolite can inhibit the RNA-dependent RNA polymerase activity of the KFDV. Dasabuvir, on the other hand, does not demonstrate detectable inhibitory activity. Further studies have been conducted to optimize the conditions and determine the inhibitory concentrations of these drugs [29].

Molecular docking studies and computer-assisted drug designing (CADD) methodologies have been suggested as an alternative to explore and repurpose the drugs to tackle KFD. These studies have potentially identified some drugs that can be repurposed to treat patients suffering from KFD [53,54].

The United States Food and Drug Administration (USFDA) approved drugs like sofosbuvir and dasabuvir which were positively evaluated for their activity against RNA-dependent RNA polymerase of NS5 protein from the KFD virus. These drugs could potentially inhibit the virus, and similar strategies may be explored further to discover drugs specifically targeted against the KFDV [55]. Anti-ED III-based neutralizing antibodies were effective in treating KFD as evidenced by the results obtained from animal experiments using mice [56].

Since 1990, in all KFD endemic areas of Karnataka, the local/state government has initiated a vaccination campaign using the formalin-inactivated tissue-culture vaccine. The vaccine was administered to all persons aged 7-65 years, subcutaneously in two doses (zero and one month) at different concentrations in adults (1 ml) and children (0.5 ml). A booster dose at 6-9 months or one year confers satisfactory protection [29]. The age group of 0-6 years was exempted from vaccination, and vaccination was recommended before the start of the rainfall/season which favors vector multiplication. 

Vaccine efficacy was low with 4% (0-96%) and 67% (0-96%), after the first and second doses, respectively [57]. A booster third dose increases the vaccine efficacy to 82.9% [9]. There is an urgent need to understand the validity of the current vaccination schedule and look for reasons for poor vaccine effectiveness [9].

Despite vaccination, people were found susceptible to infection. The recurrences of KFDV may be controlled with improvements in vaccination strategies and containing the spread of the virus. Other strategies proposed included the control of the spread of the virus between vector to animal and vector to human. Edible banana-based vaccines could be ingested by animals and humans [58]. Envelope, NS1, and NS5 protein-based subunit vaccines are being explored to develop and use against KFD [59].

Vaccine candidates synthesized on a vesicular stomatitis virus (VSV) platform wherein Ebola virus (EBOV) glycoprotein was used as a vehicle vector to carry KFD precursor membrane (prM) and envelope (E) proteins were explored. This live attenuated vector-based vaccine showed promise in the mice experimental studies [60].

Drawbacks with vaccination strategies included dropouts, opt-outs, incomplete vaccinations, and avoiding booster doses. Some other factors that contributed to unsuccessful vaccination among people included a lack of adequate vaccine stocks, disbelief about vaccine safety, and other reasons like pain at the injection site after vaccination [61].

Recent advances

Among the several neuroinvasive viruses prevalent globally, KFD and a few other viruses were identified in India [62]. It was determined that the case reporting of KFD cases and the resultant deaths along with their sociodemographic details were not standardized and uniform throughout India. However, most infected populations ranged from 15 to 64 years of age [30]. Recent experimental studies on mice have revealed that KFDV may cause apoptosis of neurons and damage the brain tissue in the cerebellum, cerebrum, cerebral cortex, and hippocampus [63]. This further improves the understanding of the pathogenesis and neurological complications in KFD. A VSV-based single-dose vaccine made of KFDV precursor membrane and envelope proteins effectively prevented the disease among pigtailed macaques. Also, this vaccine produced antibodies effective against a variant of KFDV identified as Alkhurma hemorrhagic fever virus in Saudi Arabia [64].

Because of its complex transmission cycle involving the tick vector, domestic and wild animals like cattle and rodents, implementing a One Health approach was suggested as a better intervention to comprehensively address the issue of KFD in India [3,12,65]. In silico studies have been performed to understand and identify any targets on NS2B and NS3 proteins of KFDV that could contribute to developing therapeutic drugs [66]. CADD studies have identified the utility of epigallocatechin gallate (EGCG), a polyphenol compound extracted from tea leaves that could inhibit the NS3 helicase of KFDV [67]. KFDV envelope protein (E_KFDV_) similar to the one noticed in dengue virus (E_DENV_) was found capable of binding to the host's mannose receptors, which could further aid in developing newer vaccines [68]. Since January 2024, 2567 suspected KFD cases have been identified in Karnataka, among which 68 (2.65%) returned positive laboratory tests. Two deaths (0.08%) attributed to KFD were confirmed underscoring the importance of KFD controlling and preventive measures [69]. KFD has been identified as an emergent tropical disease with the potential for spreading to non-endemic regions. Factors contributing to the complexities of the disease include high death rates (3-15%) compared to dengue (<3%) as noticed in non-human primates. Lack of continuous supply of vaccines, social stigma, changing epidemiological patterns of the virus and clinical course of the disease, and inaccurate and inadequate data affect KFD control and preventive measures in humans [70].

## Conclusions

The pathogenesis of KFD appears to be complex and remains poorly understood. Monkeys amplify the virus, while cattle act as maintenance hosts. Small forest mammals can also maintain and infect ticks which continue the wild cycle helping the virus to remain viable. No specific treatment or cure is available for KFD, and the available vaccine's efficacy is limited. Prevention involves tick control through acaricides, repellents, and vegetation transformations. Tick management is crucial for lowering bite risk to humans and animals. Supportive treatment with hydration and blood products is key due to the lack of specific antiviral drugs. Sofosbuvir and its metabolite offer the potential to inhibit viral activity. Spread of the virus to non-endemic geographical regions should be considered a cause for serious concern. Seroprevalence studies in humans, domestic, and wild animals and molecular analysis of tick populations for the presence of KFDV may increase the understanding of the biology, ecology, and transmission dynamics. Further research is required to develop newer therapeutic drugs and efficient vaccines to control and prevent the disease.
